# Development and psychometric evaluation of public stigma of stroke scale (PSSS)

**DOI:** 10.1038/s41598-023-27504-8

**Published:** 2023-01-11

**Authors:** Meijuan Wan, Yibing Tan, Yimin Huang, Qishan Zhang, Fengyin Qin, Xinglan Sun, Fen Wang, Jia Wang, Xiaopei Zhang

**Affiliations:** 1 School of Nursing, Xi’an Jiaotong University Health Science Center, Xi’an, China; 2 The Second Clinical College of Guangzhou University of Chinese Medicine, Guangzhou, Guangdong China; 3 School of Nursing, Guangzhou University of Chinese Medicine, No. 232, Waihuan East Road Higher Education Mega Center, Guangzhou, Guangdong China; 4 Neurology Department, The Second Affiliated Hospital of Guangzhou University of Chinese Medicine, Guangzhou, Guangdong China

**Keywords:** Psychology, Diseases, Health care

## Abstract

Stroke patients suffer from public stigma because strokes cause visible disability and heavy social burden. However, existing tools measuring stroke-related stigma do not consider public stigma. The aim of this study was to develop and evaluate a public stigma of stroke scale (PSSS). This cross-sectional study recruited 730 participants, aged above 18 years, with no diagnosis of stroke before. Scale items were generated after reviewing relevant literature and conducting interviews. An expert panel evaluated the validity and reliability of a preliminary scale. Exploratory factor analysis (EFA) and confirmatory factor analysis (CFA), bifactor CFA (B-CFA), Exploratory structural equation modelling (ESEM), bifactor-ESEM (B-ESEM) were performed to extract factors and evaluate fit on the factor structures. The Omega coefficient was 0.93, and the test–retest reliability coefficient was 0.721. The EFA extracted four factors: inherent ideology, aesthetic feelings, avoidance behaviour, and policy attitudes. These explained 61.57% of the total variance in the data. The four-factor model was confirmed by B-CFA, and met the fitness criteria. The PSSS yields satisfactory psychometric properties and can be used to assess stroke-related public stigma.

## Introduction

Stroke is the second-leading cause of death and the third-leading cause of combined death and disability in the world^1^. In China, it is the leading cause of death and disability^2^. Stroke survivors often suffer from varying degrees of disability^3^. The 12-month disability rate was 16.6% for stroke survivors in China^4^. Walking dysfunction occurs in more than 80% of stroke survivors^5^. Consequently, gait impairments cause difficulties in performing activities of daily living and mobility—Post-stroke gait is characterised by a pronounced clinical presentation of gait asymmetry^6,7^. Strokes lead to long-lasting disability and have a social impact. The lives of survivors and their families are strongly influenced by these long-term consequences, including physical disability, cognitive disorders, difficulty in concentration or some other severe psychological problems^8–10^.

Stigma is an attribute that discredits a person, reducing them ‘from a whole and usual person to a tainted, discounted one’^11^. Health-related stigma refers to the stigmatisation of disease^12^. It is characterised by exclusion, rejection, blame or devaluation that results from experience, perception or reasonable anticipation of an adverse social judgment about a person or group^13^. In 2002, stigma was divided into two types: self- and public stigma^14^. Self-stigma is formed by the integration and internalisation of public stigma. When severe, it generates negative emotions such as shame, low self-esteem and even suicide. Public stigma is distinguished from self-stigma as the reaction that the general population has to people with disease, caused by stereotypes, prejudice, and discrimination^14^.

Goffman mentioned that stigma is a phenomenon that causes social devaluation or social suspicion due to a sign or attribute^11^. Patients of acquired immune deficiency syndrome (AIDS), mental illness, cancer and chronic diseases suffer public stigma because of the infectiousness, danger, lethality, attribution, and destructive nature of the disease^15–18^. For stroke patients, this attribute or sign is the disease itself. Perceptions of stigma have been identified as a concern among stroke patients. Patients sometimes feel avoided by others^19^. Work exploring stroke-related stigma has focused on stroke patients at different stages of their rehabilitation or looked at stroke as a chronic disease^20,21^.

There are some tools that examine public stigma, such as the Cancer Stigma Scale^17^, the AIDS Attitude Scale^22^, and the Community Attitudes to Mental Illness scale^23^. However, few studies have explored stroke-related stigma in the non-patient population as extension of perceived stigma sources from stroke patients^19,24–26^, although there are several reasons why this is important. The availability of stroke screening and prevention strategies (e.g. screening high-risk community populations and health education programs) means that people need to consider the possibility of a stroke diagnosis. However, fear of stigmatisation has been identified as a potential barrier to high-risk population screening, and to carry out public health education^17^.

This study aims to develop a scale measuring stroke-related stigma in non-patient populations. Being able to measure stroke-related public stigma would help identify the extent to which stigma exists, monitor changes in perceptions of stroke as a result of public health education and identify risk factors for more stigmatised beliefs.

## Methods

### Study design and sample

**Figure 1:**
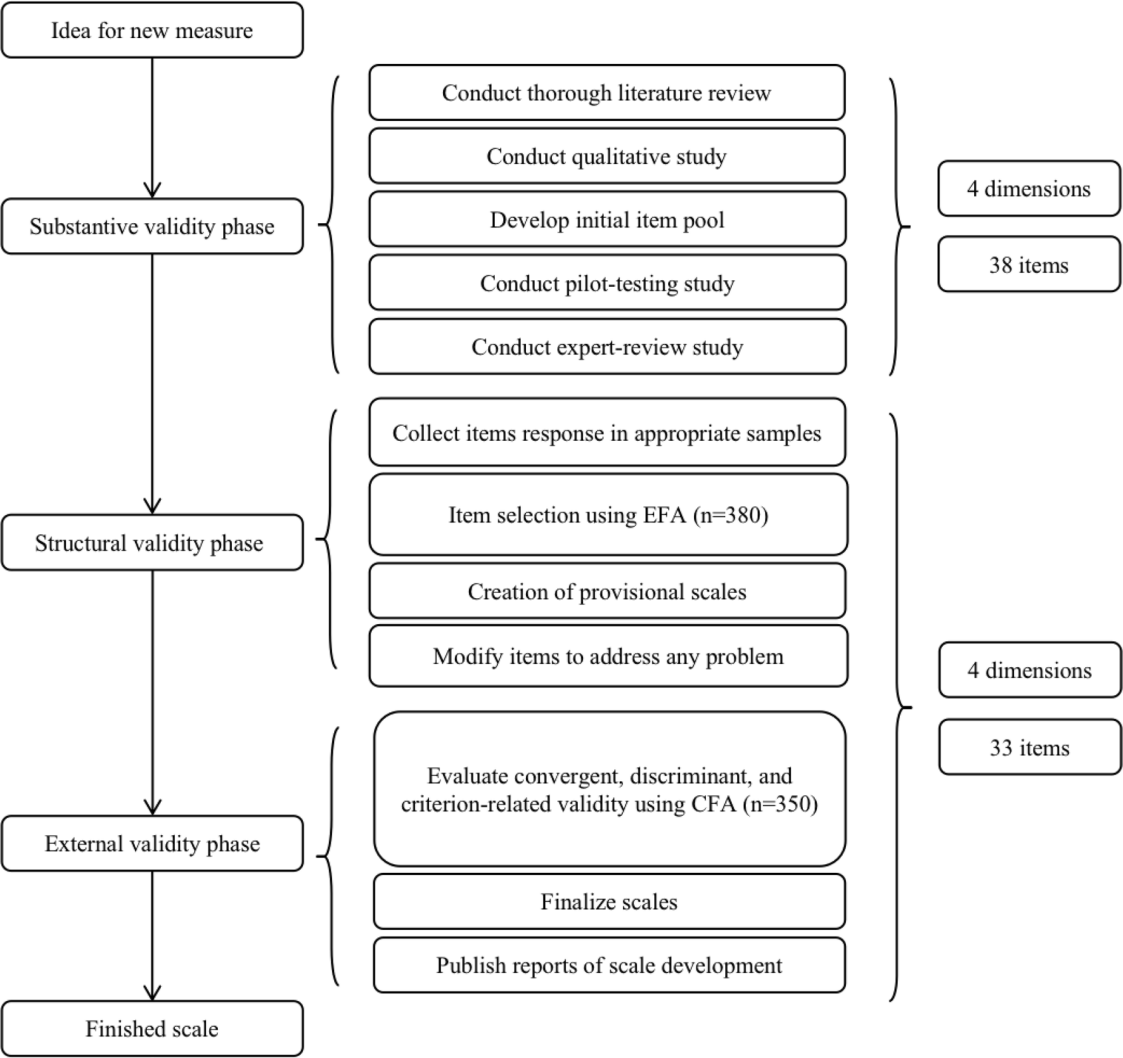
The instrument development and evaluation. PSSS, public stigma of stroke scale; EFA, exploratory factor analysis; CFA, confirmatory factor analysis.

A cross-sectional descriptive study design was used to test and evaluate the validity and reliability of the public stigma of stroke scale (PSSS). Figure 1 shows the flow chart of instrument development and evaluation. We calculated sample size based on evidence from the literature. According to the calculation recommendation, the sample size should be 10 times the item size^27^. The sample size for confirmatory factor analysis (CFA) should be at least 200^28^. Therefore, the sample size of 730 was qualified—380 for exploratory factor analysis (EFA), and 350 for CFA.

Participants were recruited from communities in China, using online and face-to-face method. Snowballing sampling was used for online data collection, and qualitative study. To ensure quality and completeness of the questionnaire, the duplication of responses was avoided by restricting IP addresses and ensuring that all questions were complete before submission. The inclusion criteria were as follows: (1) aged over 18 years; (2) have heard about stroke; (3) never been diagnosed with stroke; and (4) agreement to participant in the study. Individuals who (1) have been diagnosed with a stigmatised disease, such as cancer, AIDS, mental disease; and (2) experience obstacles in communication, reading, writing, and comprehension, were excluded.

### Procedure

#### Item generation

Multiple steps were utilised to generate scale items. First, a multidimensional concept of stigma theory was employed^29^, which identified six components of health-related stigma, applicable to varying degrees depending on the illness of interest. The definition of stigma served as a guide for the development of items. The first three components, ‘concealability’ (whether an illness can be hidden from others); ‘aesthetics’ (described as a primitive response by the perceiver towards a non-concealable mark that makes the person less ‘pleasing to the eye’) and ‘disruptiveness’ (whether an illness disrupts usual interactions). These three components are related to stroke patients. Stroke leaves visible legacy symptoms, such as physical impairment and language disability, which are harmful for usual interactions, and makes an individual ‘unpleasing to the eye’. The fourth component is ‘origin’, which related to when and how the disease is believed to have come about. Perceived responsibility is a particularly relevant aspect, because when a patient is believed to have caused their disease, the associated stigma is greater^29,30^. Stroke patients’ bad habits, such as smoking and drinking problems, make this component relevant to stroke-related stigma. The last two components are ‘peril’ (relates to perceived danger from the stigmatised person; for example, some diseases are considered contagious or dangerous, such as HIV or Mental illnesses) and ‘course’ (refers to changes in the disease over time; for example, chronic and incurable diseases are more stigmatised). These six components highlight the aspects of a disease that may contribute to it being stigmatized.

Second, we performed a comprehensive literature search for health-related stigma, existing scales and relevant items. Traditionally, besides the aspects of a disease that may stigmatized, studies also considering behavioural aspects of stigma such as interpersonal avoidance and social distance, and attitudes towards discrimination such as employment law. Therefore, we developed items basis on previous research into health-related stigma^17,20,22,23^.

Third, to better structure the appropriate items, community residents (*n* = 28) were interviewed using semi-structured open-ended interviewing methods. Participants shared their views, experiences and attitudes when they come in contact with stroke patients. The interview outlines were: (a) Have you been in contact with a stroke patient? How were they? How did you feel during these specific experiences? (b) What do you think are the causes of stroke? (c) What do you think about the quality of life of stroke patients? (d) Do you know about the long-term disabilities caused by stroke? What is the impact of these disabilities? (e) How do you feel about stroke patients with disabilities? (f) Do you think stroke patients should be treated as different people? (g) Do you think stroke is caused by the patient’s own mistakes?

Content-based analysis was conducted; two researchers reviewed the transcript and independently coded the main themes to guarantee reliability and dependability. Three themes were generalised in the analysis: unconcealable appearance, stereotypes, and attitude towards stroke patients. The results of content-based analysis support the idea that not only the stigmatizing properties of the disease itself should be considered, but also stigmatizing behaviours and attitudes. To sum up the results of the qualitative study, the multidimensional concept of stigma theory and literature review, subsequently, 49 items were generated across five dimensions: perception of stroke, aesthetics, attributes, communication, and public policy. The first three dimensions were considered as the aspects that may be stigmatized by the disease itself, and the last two dimensions were considered as stigmatizing behaviours and attitudes respectively.

#### Testing version of PSSS

Expert consultation was adopted to revise the item pool and a pilot study was conducted to certify the testing version of PSSS.

Nursing professors with extensive experience in stroke and stigma, anthropologists, psychologists, and community experts who have experiences in related research, have a title of associate professor or above, were grouped into an expert panel (*n* = 7). Expert panel members rated each item’s relevance based on three criteria using a four-point Lynn scale (1 = not at all relevant; 2 = slightly relevant; 3 = fairly relevant; and 4 = very relevant)^31^: the appropriate item attribution; dimensional saturation; and item expression accuracy. The Item content validity index (I-CVI) and the Scale content validity index (S-CVI) were calculated^32^. For each item, the I-CVI was computed as the number of experts who provided a rating of 3 or 4, divided by the total number of experts, the proportion in agreement about relevance. We used two approaches to calculate S-CVI. First, to require universal agreement among experts, defining the S-CVI as the proportion of items on an instrument that achieved a rating of 3 or 4 by all content experts. Second, computing the I-CVI for each item on the scale, and then calculating the average I-CVI across items. An I-CVI of 0.78 or higher for three or more experts could be considered evidence of good content validity^32^. The universal agreement of S-CVI should be 0.80 or higher^33^, and the average S-CVI should be 0.90 or higher^34^.

Subsequently, in the first round of expert consultation, the CVI was calculated for each item. The results indicate that I-CVI of 12 items was under 0.78; universal S-CVI was 0.43 which should be 0.80 or higher; average S-CVI was 0.86 which should be 0.90 or higher; 1 item was suggested to be deleted due to repeated expression; 2 items (perception of stroke and communication) were suggested to be modified for not being specific enough; and 2 items were suggested to be added in the communication dimension. As for the dimension of attribute only remained 2 items which cannot support a single dimension, then the 2 items were contained in the dimension of perception of stroke based on the semantics. Thus, 38 items across 4 dimensions (perception of stroke, aesthetics, communication, and public policy) were retained. And then, we conducted second round expert consultation which contains 5 experts. The results indicate that I-CVI was 0.8–1; universal S-CVI was 0.92, average S-CVI was 0.98. Therefore, there was no revision for the second round expert consultation.

We conducted a pilot test to assess the test–retest reliability. Data was collected from 52 participants to complete the pilot test twice a week apart^35^. No items required modification.

#### Psychometric evaluation

Validity (face, content, construct) and reliability (McDonald’s Coefficient Omega, and test–retest) were evaluated. To assess the face validity, items were checked for wording, order arrangement, degree of understandability and difficulty with 12 community residents. No items required modification. Item analysis was associated with the pooled items among construct validity. In the construct validity process, the pooled potential items were reduced, and factors were extracted using EFA, and confirmed using independent cluster assumption (ICM) of CFA, bifactor-CFA (B-CFA), exploratory structural equation modelling (ESEM) and bifactor-ESEM (B-ESEM).

### Measurements

#### Socio-demographic and health status

Community residents completed a self-administered questionnaire on age, sex, education level, profession, residential area, medical background, health status (such as hypertension, diabetes, heart diseases, cancer, mental illness), awareness about stroke, contact with stroke patients, and to understanding of stroke.

#### Testing version of PSSS

The PSSS was designed as a five-point Likert scale (1 = completely disagree, 2 = somewhat disagree, 3 = neither agree nor disagree, 4 = somewhat agree, and 5 = completely agree). There were 4 dimensions and 38 items in testing version of PSSS.

### Ethical considerations

The study protocol was approved by the Ethics Committee of Guangdong Provincial Hospital of Chinese Medicine. Participation was voluntary, the informed consent was set at the top of the questionnaire, participants anonymously answered the questions that were consistent with their agreement to participate, and agreed to the publication of the results. The investigation conforms with the principles outlined in the Declaration of Helsinki (Br Med J 1964;ii:177).

### Data analysis

All data analyses were conducted using SPSS version 24 and Mplus version 8^36^. Descriptive statistics were used to summarise the data.

#### Reliability

The internal consistency reliability was tested using McDonald’s Coefficient Omega. A score above 0.80 was considered to be a satisfactory measure^37^. Test–retest reliability was measured using intraclass correlation coefficients. Reliability coefficients above 0.70 were considered satisfactory^38^.

*Construct validity* The Kaiser–Meyer–Olkin (KMO) test and Bartlett’s test of sphericity were used to ensure the adequacy of data for EFA. The Bartlett’s test of sphericity must be significant (*p* < 0.05) and the KMO value must be higher than 0.60^39^. Parallel analysis and Velicer’s minimum average partial (MAP)^40^ were employed to determine the number and stability of factor extraction^41^. A minimum squares with oblimin rotation was conducted^42^. Items loading at approximately 0.50 on one dimension and not showing cross-loading above 0.45 were retained for analysis in the model^43^.

ICM-CFA, B-CFA, ESEM, B-ESEM were performed using MLR^44,45^ to evaluate the fitness on the assumed theoretical dimensions of the PSSS. In ICM-CFA, each indicator is assumed to correspond to a single factor, and B-CFA explicitly accommodates psychometric multidimensionality in the indicators by relaxing the ICM-CFA. Assessing conformations of stratified tissues requires a bifactor model, whereas assessing conceptually adjacent conformations requires an ESEM. However, the bifactor model may express unmodeled cross-loadings through inflated G-factors, whereas the ESEM model may express unmodeled G-factors through inflated cross-loadings^46^. The goodness of fit criteria range was calculated using adjusted chi-square (χ^2^/*df*), comparative fit index (CFI > 0.95), Tucker-Lewis index (TLI > 0.95), root mean square error of approximation (RMSEA ≤ 0.06)^47^.

### Ethical approval and consent to participate

The study protocol was approved by the Ethics Committee of Guangdong Provincial Hospital of Chinese Medicine. Participation was voluntary, the informed consent was set at the top of the questionnaire, participants anonymously answered the questions that were consistent with their agreement to participate, and agreed to the publication of the results.

## Results

### Demographics and health status

Participants included 730 individuals, over the age of 18, who had not been previously diagnosed with stroke or other well stigmatised diseases. Table 1 shows the participants’ demographic data. The mean age of the participants was 39.23 ± 14.59 years, ranging from 18 to 86 years.

**Table 1:** Participants’ demographics (N = 730).

Characteristics	Mean ± SD or *n* (%)
**Age, years**	39.23 ± 14.59
18–25	201 (27.5)
26–35	113 (15.5)
36–45	138 (18.9)
46–55	170 (23.3)
56–65	93 (12.8)
66–75	10 (1.4)
76–86	5 (0.7)
Sex: Male	250 (35.5)
**Education**	
Below elementary school	10 (1.4)
Middle school	54 (7.4)
High school	154 (21.1)
Bachelor’s degree	401 (54.9)
Master’s degree	99 (13.6)
Doctoral degree	12 (1.6)
**Profession**	
Farmer	21 (2.9)
Worker	56 (7.7)
Institutional personnel	202 (27.7)
Medical staff	47 (6.4)
Public service staff	19 (2.6)
Retiree	71 (9.7)
Student	175 (24.0)
Others	139 (19.0)
Medical background	97 (13.3)
**Residential area**	
Provincial capital or municipality	156 (21.4)
Prefecture-level city	315 (43.2)
County or village	259 (35.5)
**Health status**	
Healthy	693 (94.9)
Hypertension	7 (1.0)
Diabetes	2 (0.3)
Atrial fibrillation or heart disease	3 (0.4)
Have contact with stroke patients	385 (52.7)
**Extent of understanding the stroke**	
Very	108 (14.8)
A little	475 (62.6)
Not at all	165 (22.6)

### Construct validity

#### Exploratory factor analysis

The result of Bartlett’s test of sphericity was significant (χ^2^ = 8260.598, df = 406, *p* < 0.001) and the KMO value was 0.92. Parallel analysis and Velicer’s MAP showed that the four-factor model had stability of factor extraction (Supplemental Tables 1 and 2). EFA was undertaken using minimum squares with an oblimin to determine the underlying dimensions of the PSSS. Four factors were extracted, with loadings of 0.4 or greater (Table 2): factor 1, inherent ideology, refers to stigma generated by the stereotype; factor 2, aesthetic feelings, refers to a feeling generated from stroke patients’ appearance; factor 3, avoidance behaviour refers to stigmatized behaviour; and factor 4, policy attitudes refer to stigmatized attitudes. These four extracted factors explained 61.57% of the total variance in stroke-related public stigma. Therefore, 33 items in a four dimensions scale were generated.

**Table 2:** Exploratory factor analysis with the extraction of four factors (N = 380).

Items	Factor loadings			
	Factor 1	Factor 2	Factor 3	Factor 4
1. Stroke is a lifelong disease (such as disability)	**0.468**	− 0.072	− 0.042	− 0.042
2. Most stroke patients are unable to do their previous job	**0.628**	− 0.008	− 0.073	− 0.019
3. Stroke will destroy one’s self-care ability	**0.725**	− 0.026	− 0.072	0.036
4. I would feel embarrassed if I had a stroke	**0.496**	0.034	0.109	0.036
5. A stroke is worse than death	**0.615**	− 0.016	0.113	0.032
6. Stroke destroys intimate relationships	**0.667**	0.114	0.102	− 0.041
7. Stroke is a huge disaster for individuals and families	**0.729**	0.019	− 0.041	− 0.062
8. Stroke is an unlucky thing	**0.501**	0.096	0.149	0.077
9. Stroke is a serious social burden	**0.560**	0.177	0.044	− 0.019
10. The social status of stroke patients will be significantly reduced	**0.624**	0.061	0.105	0.017
14. Stroke patients always have a crooked mouth; that makes me feel uncomfortable	0.042	**0.797**	0.031	− 0.032
15. Stroke patients always tilt their head to one side; that makes me feel uncomfortable	− 0.015	**0.896**	0.029	− 0.001
16. The stiff facial expression of stroke patients makes me feel uncomfortable	− 0.025	**0.937**	0.024	− 0.011
17. The stroke patients curled up on the bed make me feel uncomfortable	− 0.024	**0.937**	− 0.009	− 0.003
18. The pipes (such as stomach tube, urinary tube) on the stroke patients make me feel uncomfortable	0.052	**0.883**	− 0.041	0.037
19. I would deliberately avoid eye contact with stroke patients	0.097	0.284	**0.553**	0.014
20. I feel uncomfortable when I get along with stroke patients	0.102	0.322	**0.547**	− 0.008
22. I do not have the patience to talk with stroke patients	0.032	0.103	**0.721**	− 0.020
23. I would try to stay away from stroke patients	0.037	0.143	**0.716**	− 0.033
24. I do not want to be friends with stroke patients anymore	− 0.046	0.018	**0.877**	− 0.049
25. I do not want to talk about stroke with others	0.098	0.000	**0.572**	0.074
27. I do not want to continue to associate with families with stroke patients	− 0.102	− 0.083	**0.911**	− 0.012
28. I do not want to continue to associate with caregivers of stroke patients	− 0.092	− 0.077	**0.899**	− 0.016
29. Stroke patients should avoid participating in social activities	0.060	0.006	**0.609**	0.063
30. I do not want to see anything about stroke in the media	− 0.001	0.005	**0.628**	0.065
31. Stroke patients should avoid causing trouble to others	0.151	0.058	**0.488**	0.021
32. If my family or I have a stroke, I will try to hide it from the outside world	0.049	− 0.022	**0.536**	− 0.006
33. The government and society should invest more financial support for the treatment and care of stroke	− 0.106	− 0.002	0.039	**0.855**
34. The government and society should provide more public facilities for stroke patients	− 0.108	0.016	0.049	**0.891**
35. The government and society should provide long-term care insurance for stroke patients	− 0.025	− 0.027	0.023	**0.896**
36. The government and society should give priority to the needs of stroke patients	0.019	0.035	− 0.082	**0.809**
37. The government and society should support stroke patients to return to work	0.119	0.004	− 0.027	**0.696**
38. The government and society should aid the primary caregivers of stroke patients (such as holidays, subsidies)	− 0.003	0.033	0.030	**0.776**
Kaiser–Meyer–Olkin (KMO)	0.922			
Bartlett’s test of sphericity	Χ^2^ = 8981.284, df = 528, *p* < 0.001			

Factor 1, inherent ideology; Factor 2, aesthetic feelings; Factor 3, avoidance behaviour; Factor 4, policy attitudes. Factor loading > 0.4.

#### Confirmatory factor analysis

**Figure 2:**
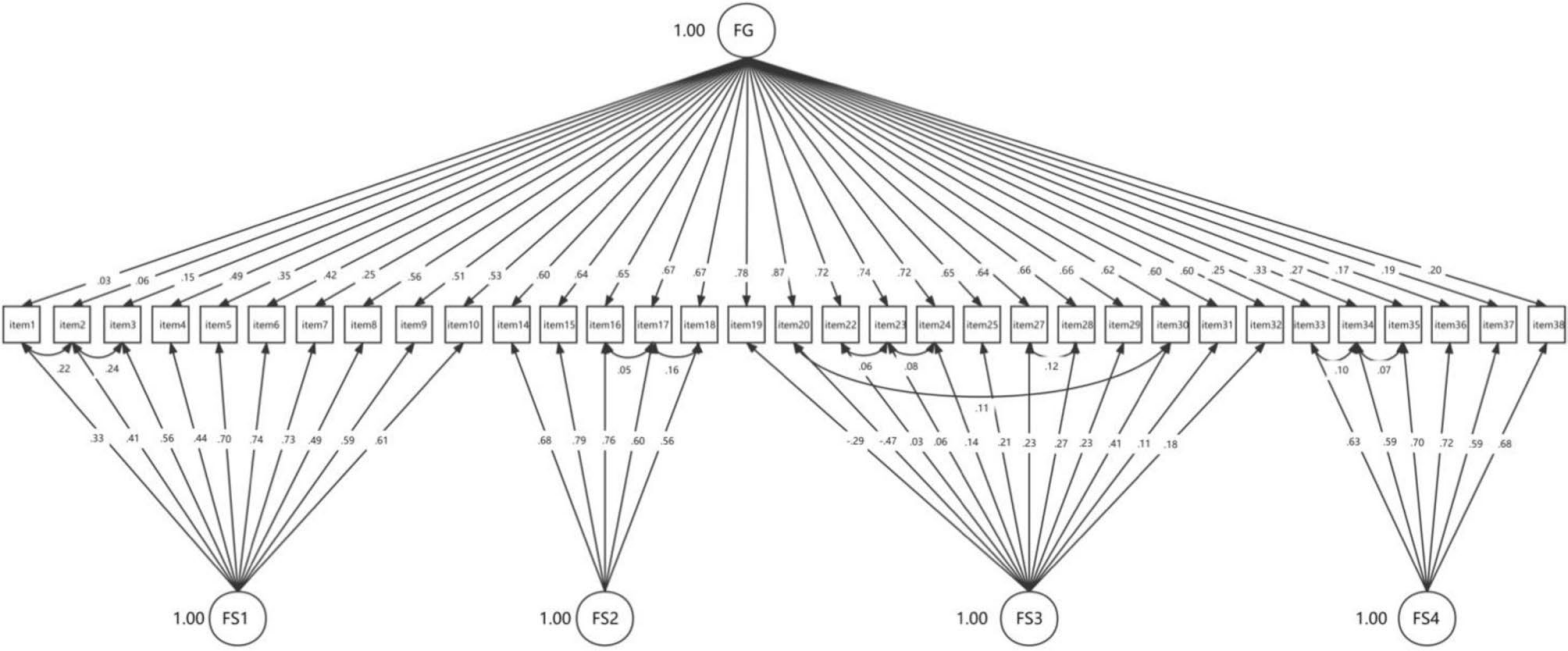
Bifactor-CFA. The factor structure of the PSSS (N = 350). Model fit index: MLR chi-square χ^2^(*df)* = 706.597 (452); comparative fit index (CFI) = 0.957; Tucker-Lewis index (TLI) = 0.950; root mean square error of approximation (RMSEA) = 0.040. FG, Global factor from a bifactor model; FS1, inherent ideology; FS2, aesthetic feelings; FS3, avoidance behaviour; FS4, policy attitudes.

ICM-CFA, B-CFA, ESEM and B-ESEM were performed to evaluate the fit on the assumed theoretical dimensions of the PSSS. The fit indices of the models were reported in Table 3. The results showed that ICM-CFA provided an unacceptable level of fitness (CFI < 0.90, TLI < 0.90), and ESEM provided a qualified CFI (0.903) but the TLI was less than 0.90. The fit indices of B-CFA and B-ESEM both showed an acceptable level of fitness but not excellent enough. So we modified the models based on modifications indices. The results suggested that the B-CFA provided the best fit to the data with CFI = 0.957, TLI = 0.950, RMSEA = 0.040. (Fig. 2). We performed 10 correlations between residuals on the theoretical basis, which were item 1 and item 2, item 2 and item 3, As a thinking of stroke is a lifelong disease such as disability, and then it will remind one feel that the stroke patients are unable to work anymore, and then they can no longer take care of themselves anymore; item 16 and item 17, item 17 and item 18, the stiff facial expression and curled up on the bed of stroke patients were disease related experiences and can make others feel uncomfortable, and then they may suffer with urinary tube etc.; item 20 and item 30, item 22 and item 23, item 23 and item 24, item 27 and item 28, when a person is reluctant to engage with a stroke patient, they will also be reluctant to see information about it on media. If a person does not have the patience to communicate with a stroke patient will also try to stay away from the stroke patient, so that in the end they cannot be friends with stroke patients, and the attributes of the family of the stroke patient and the caregiver of the stroke patient are related in that they both have close contact with the stroke patient. item 33 and item 34, item 34 and item 35, public facilities require economic investment, and public benefits and long-term care insurance are related. The parameter estimates from all models were presented in Table 4, and the correlation between factors were shown in supplementals Table 3.

**Table 3:** Goodness-of-fit statistics of the alternative measurement models.

	MLR χ^2^ (df)	CFI	TLI	RMSEA	90%CI
ICM-CFA	1226.473 (489)*	0.876	0.866	0.066	0.061, 0.070
B-CFA	954.351 (462)*	0.917	0.906	0.055	0.050, 0.060
ESEM	979.362 (402)*	0.903	0.873	0.064	0.059, 0.069
B-ESEM	759.548 (373)*	0.935	0.908	0.054	0.049, 0.060

ICM, Independent cluster model; CFA, Confirmatory factor analysis; B, Bifactor model; ESEM, Exploratory structural equation modelling; MLR, Maximum likelihood with robust standard errors; χ^2^, MLR chi square; df, Degrees of freedom; CFI, comparative fit index; TLI, Tucker-Lewis index; RMSEA, Root mean square error of approximation; CI, Confidence interval.

*p < 0.01.

**Table 4:** Standardized parameter estimates from the alternative measurement models.

	ICM-CFA		B-CFA			ESEM					B-ESEM					
	F		FG	FS		F	F	F	F		FG	FS	FS	FS	FS	
Items	λ	δ	λ	λ	δ	1λ	2λ	3λ	4λ	δ	λ	1λ	2λ	3λ	4λ	δ
**F1**																
Item1	**0.31**	0.90	**0.03**	**0.35**	0.88	**0.45**	0.07	0.14	0.02	0.85	**0.04**	**0.39**	0.02	0.03	0.04	0.84
Item2	**0.42**	0.82	**0.06**	**0.44**	0.80	**0.57**	0.07	0.13	0.10	0.73	**0.08**	**0.49**	0.02	0.01	0.16	0.72
Item3	**0.55**	0.69	**0.15**	**0.57**	0.65	**0.71**	0.01	0.21	0.03	0.59	**0.17**	**0.60**	0.06	0.08	0.08	0.59
Item4	**0.57**	0.67	**0.44**	**0.40**	0.65	**0.50**	0.08	0.25	0.01	0.64	**0.46**	**0.38**	0.03	0.06	0.05	0.64
Item5	**0.70**	0.51	**0.32**	**0.65**	0.48	**0.75**	0.01	0.05	0.06	0.49	**0.35**	**0.62**	0.07	0.03	0.04	0.49
Item6	**0.78**	0.39	**0.40**	**0.68**	0.39	**0.74**	0.04	0.02	0.00	0.41	**0.42**	**0.63**	0.11	0.01	0.09	0.41
Item7	**0.66**	0.56	**0.23**	**0.67**	0.50	**0.71**	0.06	0.14	0.01	0.52	**0.26**	**0.62**	0.12	0.03	0.12	0.52
Item8	**0.67**	0.55	**0.51**	**0.45**	0.54	**0.47**	0.08	0.25	0.00	0.54	**0.52**	**0.41**	0.11	0.13	0.05	0.53
Item9	**0.69**	0.52	**0.45**	**0.53**	0.52	**0.58**	0.02	0.21	0.01	0.54	**0.47**	**0.49**	0.05	0.07	0.07	0.53
Item10	**0.72**	0.48	**0.46**	**0.54**	0.49	**0.60**	0.09	0.11	0.03	0.50	**0.48**	**0.50**	0.12	0.06	0.05	0.49
**F2**																
Item14	**0.85**	0.28	**0.56**	**0.65**	0.27	0.03	**0.88**	0.02	0.05	0.28	**0.58**	0.08	**0.62**	0.01	0.04	0.27
Item15	**0.96**	0.08	**0.62**	**0.75**	0.05	0.02	**0.97**	0.00	0.02	0.08	**0.64**	0.11	**0.70**	0.03	0.11	0.08
Item16	**0.97**	0.06	**0.63**	**0.73**	0.07	0.02	**0.99**	0.02	0.00	0.05	**0.65**	0.11	**0.71**	0.02	0.10	0.05
Item17	**0.87**	0.25	**0.62**	**0.57**	0.30	0.07	**0.79**	0.06	0.01	0.25	**0.64**	0.14	**0.57**	0.03	0.07	0.24
Item18	**0.80**	0.36	**0.61**	**0.51**	0.37	0.12	**0.68**	0.09	0.05	0.34	**0.63**	0.16	**0.48**	0.15	0.12	0.31
**F3**																
Item19	**0.71**	0.49	**0.78**	**0.30**	0.30	0.08	0.15	**0.59**	0.04	0.48	**0.81**	0.03	0.01	**0.35**	0.04	0.22
Item20	**0.76**	0.42	**0.87**	**0.47**	0.02	0.07	0.20	**0.59**	0.01	0.41	**0.86**	0.04	0.03	**0.36**	0.02	0.14
Item22	**0.77**	0.40	**0.77**	**0.03**	0.41	0.06	0.03	**0.73**	0.01	0.41	**0.77**	0.00	0.02	**0.02**	0.04	0.40
Item23	**0.85**	0.28	**0.82**	**0.07**	0.33	0.03	0.03	**0.88**	0.08	0.27	**0.83**	0.06	0.01	**0.12**	0.01	0.29
Item24	**0.85**	0.28	**0.81**	**0.15**	0.32	0.01	0.08	**0.92**	0.02	0.26	**0.82**	0.06	0.09	**0.17**	0.06	0.28
Item25	**0.70**	0.52	**0.68**	**0.22**	0.49	0.06	0.14	**0.79**	0.08	0.49	**0.68**	0.01	0.12	**0.16**	0.01	0.50
Item27	**0.77**	0.41	**0.71**	**0.26**	0.43	0.07	0.13	**0.70**	0.06	0.40	**0.72**	0.06	0.07	**0.40**	0.09	0.30
Item28	**0.84**	0.29	**0.80**	**0.33**	0.26	0.02	0.02	**0.83**	0.11	0.27	**0.79**	0.05	0.05	**0.41**	0.16	0.17
Item29	**0.71**	0.50	**0.69**	**0.24**	0.47	0.03	0.01	**0.68**	0.02	0.50	**0.67**	0.01	0.01	**0.23**	0.06	0.50
Item30	**0.72**	0.48	**0.67**	**0.45**	0.34	0.09	0.01	**0.73**	0.13	0.45	**0.67**	0.11	0.04	**0.29**	0.18	0.42
Item31	**0.60**	0.64	**0.60**	**0.11**	0.63	0.05	0.08	**0.51**	0.03	0.64	**0.58**	0.03	0.04	**0.10**	0.05	0.65
Item32	**0.64**	0.60	**0.63**	**0.19**	0.57	0.10	0.03	**0.58**	0.06	0.59	**0.61**	0.05	0.03	**0.16**	0.09	0.60
**F4**																
Item33	**0.80**	0.36	**0.27**	**0.70**	0.44	0.03	0.02	0.01	**0.80**	0.36	**0.24**	0.06	0.04	0.06	**0.75**	0.37
Item34	**0.88**	0.23	**0.40**	**0.72**	0.33	0.03	0.06	0.10	**0.84**	0.22	**0.35**	0.10	0.02	0.11	**0.80**	0.22
Item35	**0.87**	0.24	**0.31**	**0.78**	0.30	0.00	0.03	0.03	**0.87**	0.24	**0.26**	0.10	0.09	0.05	**0.82**	0.23
Item36	**0.78**	0.39	**0.19**	**0.81**	0.31	0.01	0.04	0.06	**0.81**	0.37	**0.16**	0.11	0.09	0.03	**0.77**	0.36
Item37	**0.64**	0.59	**0.21**	**0.65**	0.54	0.02	0.02	0.01	**0.65**	0.59	**0.18**	0.06	0.06	0.01	**0.62**	0.58
Item38	**0.81**	0.34	**0.24**	**0.82**	0.27	0.01	0.01	0.02	**0.83**	0.33	**0.20**	0.08	0.06	0.07	**0.78**	0.33

Main a priori factor loadings are bolded; ICM, Independent cluster model; CFA, Confirmatory factor analysis; B, Bifactor model; ESEM, Exploratory structural equation modelling; λ, Standardized factor loading; δ, Standardized uniqueness; FG, Global factor from a bifactor model; FS, Specific factor from a bifactor model. F1, inherent ideology; F2, aesthetic feelings; F3, avoidance behaviour; F4, policy attitudes.

### Reliability

McDonald’s omega coefficient revealed the high reliability of the PSSS (McDonald’s omega = 0.93). The subscales indicated good internal consistency, with McDonald’s omega values ranging from 0.87 to 0.95. Test–retest reliability coefficient of the total scale was 0.721.

## Discussion

The present study developed the PSSS, aimed to assess stroke-related public stigma in non-patient populations. Four factors (inherent ideology, aesthetic feelings, avoidance behaviour, policy attitudes) were determined using EFA and B-CFA.

Factor 1, ‘inherent ideology,’ refers to the subjective view of non-patient populations that is generated by the stereotype of stroke patients. We designed items which indicated stroke as a chronic disease, deprivation of working ability and self-care ability, and allowing transposition thinking such as ‘I would feel embarrassed if I had a stroke,’ and ‘A stroke is worse than death.’ Stereotypes are especially efficient means of categorising information about social groups. They are considered ‘social’ because they represent collectively agreed upon notions of groups of persons. They are ‘efficient’ because people can quickly generate impressions and expectations of individuals who belong to a stereotyped group^48^. This conception is well studied in mental illness stigma^14,16^. The item ‘Stroke is a lifelong disease (such as disability)’ is similar to the item ‘getting cancer means having to mentally prepare oneself for death’ from the Cancer Stigma Scale^17^. Stroke survivors have much higher rates of hemiplegia, aphasia, dysphagia, and urinary incontinence compared with other chronic diseases. Cancer reminds of death, whereas stroke reminds of long-term disability.

Factor 2, ‘aesthetic feelings,’ implies public feelings towards visible, external manifestations among stroke patients. This factor stems from the ‘aesthetic’ dimension of Jones multidimensional concept of stigma^29^, described as a primitive response by the perceiver towards a non-concealable mark that makes the person less ‘pleasing to the eye.’ This is well confirmed in our qualitative analysis. Participants can easily describe external manifestations of stroke patients, such as tilted head, crooked mouth, and even urinary incontinence. The impression of stroke on the public is profound. However, existing tools examining stigma, fail to express this dimension^17,22,23^. Therefore, items in this dimension were generated based on qualitative data.

Factor 3, ‘avoidance behaviour,’ refers to a typical immediate response to public stigma; it is most prominent in social interaction. Stroke survivors stated that their friends or relatives become estranged after knowing the situation^13^. This is supported by our qualitative data. Participants indicated that they cannot always interact with patients and worried about causing unnecessary trouble to themselves. This may be explained through Kurzban and Leary’s theory^26^—human beings possess cognitive adaptations designed to cause them to avoid poor social exchange partners and join cooperative groups. This dimension is included in other assessment tools^49–51^. In the current study, items in this dimension involve a comprehensive social interaction category, including avoidance behaviours such as towards individuals, families, social interactions, and media.

Factor 4, ‘policy attitudes,’ refers to supportive attitudes of the public towards stroke patients. The Cancer Stigma Scale includes this dimension and has satisfactory reliability and validity^17^. Besides, the item ‘more tax money should be spent on the care and treatment of the mentally ill’ in the benevolence dimension of the Community Attitudes Toward the Mentally Ill scale, also indicated policy attitudes^23^. This study compiled positive reverse items in this dimension. Since society assumes that older adults suffer from stroke, except for treatment, support for older patients is limited. For example, the society believes that it is normal to retire at an old age; therefore, some insufficient supportive measures are taken for older stroke patients^19,24^. This dimension involves financial, public, social, and family support.

These four dimensions cover three aspects of stigma measuring: the disease itself, stigma behaviour and stigma attitude. PSSS comprehensively evaluated stroke-related public stigma. Our study developed practical and appropriate items through interviews and expert ratings; items indicated satisfactory content validity. We confirmed the four-factor model using EFA, ICM-CFA, B-CFA, ESEM and B-ESEM. Additionally, the B-CFA showed that the PSSS had a good fit index and confirmed the four-factor model. The B-CFA demonstrated that every item in the PSSS contributed to the domain of the scale; thus, the construct validity was adequate. Regarding reliability, PSSS has satisfactory internal consistency and test–retest reliability, indicating that it is consistent and stable across time.

### Limitations

Although our study confirmed that the PSSS is a valid and reliable instrument to measure stroke-related public stigma, some limitations exist. First, the study included a convenient sample, although we do ensure a wide range of age in sampling stage. Second, although we tried to avoid including items that may set social expectations, we could not avoid this situation. Third, the current study assessed validity and reliability in a Chinese context, further studies would be needed to evaluate public stigma of stroke in other contexts. Fourth, the classical test theory has been criticised by scholars of modern measurement theory because of the “inherent flaws” of the mathematical model on which it is based, and there is an urgent need to test the PSSS against modern measurement theory, for example by using Item Response Theory to test the difficulty and discrimination of the PSSS items, which is another important direction for future scale development and application.

## Conclusions

Stroke-related stigma could have a negative influence on both, patients, and non-patient populations. Our study demonstrated that the PSSS is an appropriate instrument to assess stroke-related stigma among non-patient populations. The availability of the PSSS would help assess levels of stroke-related public stigma and design interventions to decrease stigma.

## Supplementary Information

Supplementary Information.

## Data Availability

All data generated or analysed during this study are included in this published article. The datasets generated and/or analysed during the current study are available from the corresponding author on reasonable request.

## Abbreviations

PSSS: Public stigma of stroke scale
EFA: Exploratory factor analysis
CFA: Confirmatory factor analysis
B-CFA: Bifactor confirmatory factor analysis
ESEM: Exploratory structural equation modelling
B-ESEM: Bifactor exploratory structural equation modelling
AIDS: Immune deficiency syndrome
I-CVI: Item content validity index
S-CVI: Scale content validity index
KMO: Kaiser–Meyer–Olkin
CFI: Comparative fit index
TLI: Tucker-Lewis index
RMSEA: Root mean square error of approximation

## References

[1] Collaborators, G. S. (2021). Global, regional, and national burden of stroke and its risk factors, 1990–2019: A systematic analysis for the Global Burden of Disease Study 2019. Lancet Neurol..

[2] Report on Stroke, P., Treatment in China Writing, G. (2020). Brief report on stroke prevention and treatment in China, 2019. Chin. J. Cerebrovasc. Dis..

[3] Campbell BCV (2019). Ischaemic stroke. Nat. Rev. Dis. Primers.

[4] Tu WJ (2021). Case-fatality, disability and recurrence rates after first-ever stroke: A study from bigdata observatory platform for stroke of China. Brain Res. Bull..

[5] Duncan PW (2005). Management of Adult Stroke Rehabilitation Care: A clinical practice guideline. Stroke.

[6] Olney SJ, Richards C (1996). Hemiparetic gait following stroke. Part I: Characteristics. Gait Posture.

[7] Richards CL, Olney SJ (1996). Hemiparetic gait following stroke. Part II: Recovery and physical therapy. Gait Posture.

[8] Jaracz K, Grabowska-Fudala B, Górna K, Kozubski W (2014). Consequences of stroke in the light of objective and subjective indices: A review of recent literature. Neurol. Neurochir. Pol..

[9] Kamwesiga JT, Tham K, Guidetti S (2017). Experiences of using mobile phones in everyday life among persons with stroke and their families in Uganda: A qualitative study. Disabil. Rehabil..

[10] Crichton SL, Bray BD, McKevitt C, Rudd AG, Wolfe CD (2016). Patient outcomes up to 15 years after stroke: Survival, disability, quality of life, cognition and mental health. J. Neurol. Neurosurg. Psychiatry.

[11] Goffman E (1963). Stigma: Notes on the management of spoiled identity.

[12] Scambler G (2009). Health-related stigma. Sociol. Health Illn..

[13] Weiss MG, Ramakrishna J, Somma D (2006). Health-related stigma: Rethinking concepts and interventions. Psychol. Health Med..

[14] Corrigan PW (2002). The paradox of self-stigma and mental illness. Clin. Psychol. Sci. Pract..

[15] Kalichman SC (2013). The harms of internalized AIDS stigma: a comment on Tsai et al.. Ann. Behav. Med. Publ. Soc. Behav. Med..

[16] Corrigan PW (2000). Mental health stigma as social attribution: Implications for research methods and attitude change. Clin. Psychol..

[17] Marlow LA, Wardle J (2014). Development of a scale to assess cancer stigma in the non-patient population. BMC Cancer.

[18] Lu Q (2019). Reliability and validity of a Chinese version of the Stigma Scale for Chronic Illness (SSCI) in patients with stroke. Top Stroke Rehabil..

[19] Anderson S, Whitfield K (2011). An ecological approach to activity after stroke: It takes a community. Top. Stroke Rehabil..

[20] Zhu M (2019). The Stroke Stigma Scale: A reliable and valid stigma measure in patients with stroke. Clin. Rehabil..

[21] Molina Y, Choi SW, Cella D, Rao D (2013). The stigma scale for chronic illnesses 8-item version (SSCI-8): Development, validation and use across neurological conditions. Int. J. Behav. Med..

[22] Froman R, Owen SV, Daisy C (1992). Development of a measure of attitudes toward persons with AIDS. J. Sch. Nurs..

[23] Taylor SM, Dear MJ (1981). Scaling community attitudes toward the mentally ill. Schizophr. Bull..

[24] Earnshaw VA, Quinn DM, Kalichman SC, Park CL (2013). Development and psychometric evaluation of the Chronic Illness Anticipated Stigma Scale. J. Behav. Med..

[25] Omu O, Reynolds F (2012). Health professionals' perceptions of cultural influences on stroke experiences and rehabilitation in Kuwait. Disabil. Rehabil..

[26] Kurzban R, Leary MR (2001). Evolutionary origins of stigmatization: The functions of social exclusion. Psychol. Bull..

[27] Everitt BS (1975). Multivariate analysis: The need for data, and other problems. Br. J. Psychiatry.

[28] Cappelleri JC, Bushmakin AG, Harness J, Mamolo C (2013). Psychometric validation of the physician global assessment scale for assessing severity of psoriasis disease activity. Qual. Life Res..

[29] Jones EE, Farina A, Hastorf AH, Markus H, Miller DT, Scott RA (1984). Social Stigma: The Psychology of Marked Relationships.

[30] Crocker, J., Major, B. & Steele C. Social stigma. In *The Handbook of Social Psychology. 4th edition*, Vol. 2 (Academic Press, 1998).

[31] Lynn MR (1986). Determination and quantification of content validity. Nurs. Res..

[32] Polit DF, Beck CT, Owen SV (2007). Is the CVI an acceptable indicator of content validity? Appraisal and recommendations. Res. Nurs. Health.

[33] Davis L (1992). Instrument review: Getting the most from a panel of experts. Appl. Nurs. Res..

[34] Waltz CF, Strickland O, Lenz ER (2005). Measurement in Nursing and Health Research.

[35] Golay P, Favrod J, Morandi S, Bonsack C (2019). Psychometric properties of the French-language version of the Coercion Experience Scale (CES). Ann. Gen. Psychiatry.

[36] Muthén, L. K. & Muthén B. O. *Mplus User’s Guide. 8th Edition* (Muthén & Muthén, 1998–2017).

[37] McDonald RP (1999). Test Theory: A Unified Treatment.

[38] Ekstrand E, Lexell J, Brogårdh C (2018). Test-retest reliability of the Life Satisfaction Questionnaire (LiSat-11) and association between items in individuals with chronic stroke. J. Rehabil. Med..

[39] Norris M, Lecavalier L (2010). Evaluating the use of exploratory factor analysis in developmental disability psychological research. J. Autism Dev. Disord..

[40] Velicer WF (1976). Determining the number of components from the matrix of partial correlations. Psychometrika.

[41] O'Connor BP (2000). SPSS and SAS programs for determining the number of components using parallel analysis and Velicer's MAP test. Behav. Res. Methods Instrum. Comput..

[42] Sellbom M, Tellegen A (2019). Factor analysis in psychological assessment research: Common pitfalls and recommendations. Psychol. Assess.

[43] Sharkey CM, Perez MN, Bakula DM, Grant DM, Mullins LL (2019). Exploratory factor analysis of the Mishel uncertainty in illness scale among adolescents and young adults with chronic medical conditions. J. Pediatr. Health Care.

[44] Rhemtulla M, Brosseau-Liard P, Savalei V (2012). When can categorical variables be treated as continuous? A comparison of robust continuous and categorical SEM estimation methods under suboptimal conditions. Psychol. Methods.

[45] Robitzsch A (2020). Why ordinal variables can (almost) always be treated as continuous variables: Clarifying assumptions of robust continuous and ordinal factor analysis estimation methods. Front. Educ..

[46] Morin AJS, Arens AK, Tran A, Caci H (2016). Exploring sources of construct-relevant multidimensionality in psychiatric measurement: A tutorial and illustration using the Composite Scale of Morningness. Int. J. Methods Psychiatr. Res..

[47] Hu LT, Bentler PM (1999). Cutoff criteria for fit indexes in covariance structure analysis: Conventional criteria versus new alternatives. Struct. Equ. Model..

[48] Hamilton, D. L., Gibbons, P. A., Stroessner, S. J. & Sherman, J. W. Stereotypes and language use (2016).

[49] Shrum JC, Turner NH, Bruce KE (1989). Development of an instrument to measure attitudes toward acquired immune deficiency syndrome. AIDS Educ. Prev..

[50] Angermeyer MC, Matschinger H (1996). The effect of personal experience with mental illness on the attitude towards individuals suffering from mental disorders. Soc. Psychiatry Psychiatr. Epidemiol..

[51] Angermeyer MC, Matschinger H (2003). The stigma of mental illness: Effects of labelling on public attitudes towards people with mental disorder. Acta Psychiatr. Scand..

