# Supplementary material for: Development and psychometric evaluation of public stigma of stroke scale (PSSS)
Source: Sci Rep. 2023 Jan 11;13:545. doi: 10.1038/s41598-023-27504-8 (PMC9834381; doi:10.1038/s41598-023-27504-8)
Supplement: Supplementary file 1 — Supplementary Information. [file 41598_2023_27504_MOESM1_ESM.pdf]

Development and Psychometric Evaluation of Public Stigma of Stroke Scale (PSSS)

Meijuan Wan, Yibing Tan, Yimin Huang, Qishan Zhang, Fengyin Qin, Xinglan Sun, Fen Wang, Jia Wang, Xiaopei Zhang

Supplementals information

Supplemental Table 1 Parallel analysis results

Root	Raw data	Means	Percentiles
1.00	11.9569	1.6561	1.73685
2.00	5.3378	1.5767	1.6345
3.00	2.5596	1.5163	1.5622
**4.00**	**1.8537**	**1.4679**	**1.5085**
5.00	1.3159	1.4216	1.4593
6.00	1.2170	1.3804	1.4182
7.00	1.1013	1.3407	1.3751
8.00	0.9034	1.3040	1.3751
9.00	0.8442	1.2714	1.3029
10.00	0.8083	1.2386	1.2688
11.00	0.7337	1.2067	1.2359
12.00	0.7043	1.1753	1.2047
13.00	0.6362	1.1458	1.1725
14.00	0.5978	1.1165	1.1429
15.00	0.5918	1.0892	1.1147
16.00	0.5487	1.0614	1.0860
17.00	0.5153	1.0343	1.0601
18.00	0.4799	1.0083	1.0335
19.00	0.4598	0.9817	1.0051
20.00	0.4190	0.9561	0.9799
21.00	0.4168	0.9315	0.9549
22.00	0.3840	0.9072	0.9314
23.00	0.3559	0.8831	0.9062
24.00	0.3435	0.8587	0.8820
25.00	0.3300	0.8347	0.8586
26.00	0.3121	0.8119	0.8355
27.00	0.2903	0.7874	0.8099
28.00	0.2885	0.7638	0.7851
29.00	0.2457	0.7404	0.7636
30.00	0.2450	0.7167	0.7388
31.00	0.2038	0.6928	0.7159
32.00	0.1936	0.6686	0.6924
33.00	0.1681	0.6443	0.6678
34.00	0.1594	0.6191	0.6438
35.00	0.1475	0.5925	0.6185
36.00	0.1418	0.5653	0.5914
37.00	0.1043	0.5355	0.5638
38.00	0.0858	0.4978	0.5313

Supplemental Table 2 Velicer (Minimum Average Partial, MAP) results

Root	Squared	Power 4
0.00	0.1100	0.0296
1.00	0.0388	0.0075
2.00	0.0258	0.0025
3.00	0.0187	0.0018
**4.00**	**0.0132**	**0.0007**
5.00	0.0135	0.0007
6.00	0.0137	0.0007
7.00	0.0130	0.0009
8.00	0.0142	0.0010
9.00	0.0151	0.0010
10.00	0.0160	0.0012
11.00	0.0184	0.0015
12.00	0.0204	0.0019
13.00	0.0226	0.0024
14.00	0.0244	0.0029
15.00	0.0270	0.0036
16.00	0.0300	0.0039
17.00	0.0332	0.0046
18.00	0.0365	0.0062
19.00	0.0405	0.0073
20.00	0.0455	0.0083
21.00	0.0516	0.0110
22.00	0.0582	0.0133
23.00	0.0666	0.0164
24.00	0.0748	0.0203
25.00	0.0878	0.0270
26.00	0.0974	0.0321
27.00	0.1102	0.0388
28.00	0.1376	0.0579
29.00	0.1704	0.0802
30.00	0.1402	0.0499
31.00	0.1564	0.0582
32.00	0.1801	0.0777
33.00	0.2249	0.1060
34.00	0.2569	0.1325
35.00	0.3411	0.2096
36.00	0.4879	0.3601
37.00	1.0000	1.0000

Supplemental Table 3 Correlations between factor of the alternative measurement models

	**ICM-CFA**				**B-CFA**			**ESEM**				**B-ESEM**		
	F2	F3	F4	G	F2	F3	F4	F2	F3	F4	G	F2	F3	F4
F1	0.58	0.51	-0.02	0.00	0.00	0.00	0.00	0.54	0.43	-0.07	0.00	0.00	0.00	0.00
F2		0.63	0.06	0.00		0.00	0.00		0.62	0.04	0.00		0.00	0.00
F3			0.39	0.00			0.00			0.36	0.00			0.00
F4				0.00							0.00			

ICM: Independent cluster model; CFA: Confirmatory factor analysis; B: Bifactor model; ESEM: Exploratory structural equation modelling; G: Global factor from a bifactor model; F1: inherent ideology; F2: aesthetic feelings; F3: avoidance behaviour; F4: policy attitudes.
